# A Rapid Method to Rank Sound Sources in a Reverberant Environment

**DOI:** 10.3390/s26175616

**Published:** 2026-09-04

**Authors:** Lakshmikanthan Chinnasamy, K. I. Ramachandran

**Affiliations:** Department of Mechanical Engineering, Amrita School of Engineering, Amrita Vishwa Vidyapeetham, Coimbatore 641112, India; c_lakshmik@cb.amrita.edu

**Keywords:** sound source ranking, multi-sound source system, dynamic mode decomposition (DMD), very-near Field (VNF), quick ranking

## Abstract

Sound generated by an engine is one of the major contributors to an automobile’s overall sound pressure level (SPL). During its development, the engine’s SPL is monitored frequently for compliance. If the SPL is found to be exceeded, sources are identified for modification. As the timeline is critical, a rapid methodology is required to reduce it. The proposed methodology employs dynamic mode decomposition (DMD), a data-driven approach for source ranking. DMD detects changes in a variable between two successive time intervals and quantifies its rate of change. Based on the physics involved, a new interpretation for DMD’s decay factor is introduced to capture the sound signal’s nature. DMD and Short-Time Fourier Transform (STFT) are compared in this study. The STFT-based systems employ a large array of microphones to rank the sources. In this study, a single microphone and DMD are used to rank the sources. DMD utilizes far less data than the conventional STFT for source identification and ranking. Though DMD requires more memory and time than STFT, the overall time to complete the ranking of sources is very short. The results are compared with calculated values and show a negligible discrepancy between them.

## 1. Introduction

Rapid ranking of equipment sound sources during its development phase provides valuable insight to designers. Early identification of malfunctions allows them to make corrections with less cost and helps in reducing the development time. The present work focuses on one such scenario wherein an internal combustion engine (ICE) in its development stage is considered as the test specimen. The sound sources present in the engine are identified and ranked in a normal engine testbed. The sound signals, if recorded during a performance trial of an engine, eliminate the cost for a separate noise source ranking study as well as a separate anechoic chamber.

**Figure 1:**
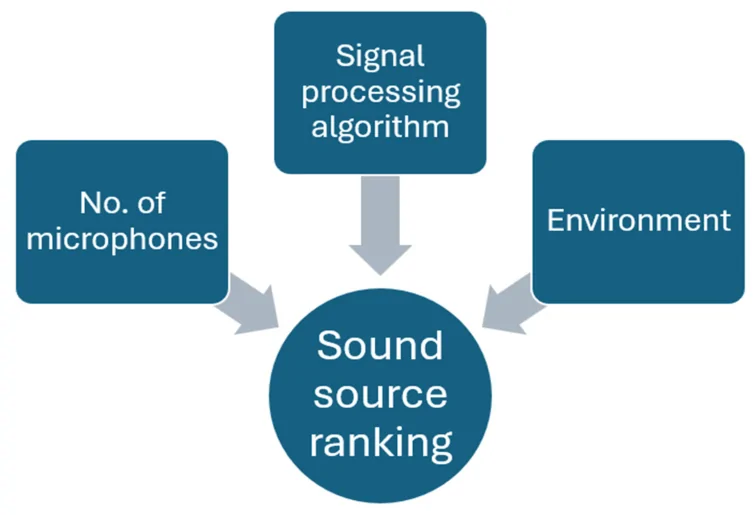
Ranking of sound sources—influencing factors.

The ranking of sound sources depends on three factors: the number of microphones, the signal processing algorithm, and the testing environment, as shown in Figure 1. The quality of a recorded signal depends on the environment. A free-field condition similar to an expensive hemi-anechoic chamber is an ideal environment for signal recording [1]. Alternatively, signals can be recorded with a low signal-to-noise ratio (SNR) in a non-anechoic environment and subsequently processed with sophisticated algorithms for source ranking. A sound signal with a signal-to-noise ratio (SNR) of 30 dB or above is essential for accurate ranking [2]. Among the various methodologies, isolation is best suited to evaluate a source’s effect on the overall sound pressure level (SPL). Masking or deactivating the source isolates it in a multi-source sound study. Source isolation by masking is expensive and time-consuming. Masking or deactivation is common in both anechoic and non-anechoic environments. Source isolation is attainable in devices with a singular source, but it is difficult in devices with multi-sound sources.

In the 1990s, John et al. [3] used sound intensity levels to rank the sources of a compressor installation and ascertain the direction in which SPL decreases. They constructed a virtual box-type control surface as a measurement plane to record sound intensity levels on all five sides of the compressor, omitting the bottom. The five faces are organized in descending order based on their sound intensity levels. These kinds of studies are predominantly used for identifying the disturbance created by the equipment. Hallman [4] developed a technique to employ near-field acoustic holography (NAH) for predicting the sound power of a source within an enclosure. Their studies accounted for the reverberations present in the environment, signifying the introduction of NAH’s use in a reverberant environment. The researchers used theoretical sound excitation models of sources to examine the sound transmission channels. Verheij et al. [5,6] utilized this methodology in conjunction with reciprocity theory to examine and rank the sound transmission paths, as studied by Ferdinand et al. [7]. These early methods measured the overall noise levels of equipment in order to assess the environmental impact. The methodologies discussed above consumed a lot of time, as defining a virtual control surface, setting up the microphone and data acquisition system, and processing the large amount of data involved considerable planning and time.

As research has progressed, innovative sensors have been developed. Iwata et al. [8] developed a new surface intensity probe and used it to assess the structure-borne noise in a refrigerator. The surface intensity probe combines an accelerometer and a microphone to directly measure the sound power from the surface. In 2000, Brüel & Kjaer (B&K) developed a next-generation sound intensity probe using two tightly aligned phase-matched microphones. Belek et al. utilized the sound intensity probe to evaluate and classify the sound sources in a household vacuum cleaner [9,10]. The B&K sound intensity probe improves resolution in sound source identification and assists in probing the sources in a multi-sound source system. The invention represents a significant advancement in sound source localization and ranking in both industrial and domestic devices [11]. The sound intensity probe reduced the time significantly. However, the cost of equipment is not affordable for every industry.

Detecting and ranking the source strength is essential for minimizing overall sound pressure levels; methodologies such as spatial transformation of sound fields (STSF), inverse boundary element methods (IBEM), and beamforming, along with near-field acoustic holography (NAH), have played a significant role in this process [12,13,14,15,16,17]. All these methods utilize either a box-shaped or body-conforming array of microphones, employing a minimum of 9 and extending up to 144 or more, to cover the entire surface of the device. As the number of microphones increases, the volume of data collected also increases proportionately, as reported by Hald et al. [18] and Johnson et al. [19]. Baars et al. [20] performed a source path contribution (SPC) study to determine the influence of a particular source on the interior. The SPC study utilized 45 accelerometer channels and 14 microphone channels to collect the data. Comesana et al. [21] used panel noise contribution analysis (PNCA) to rank the internal noise sources in an electric vehicle. The PNCA requires vibration and noise data to assess the rankings of the sources. The interior noise mapping and contribution analysis employ a total of 176 measurement locations with 11 pressure velocity (p-u) probes.

Beamforming uses large microphone arrays to collect data. Doubler [22] utilized three 5 m × 3 m array systems consisting of 576 channels to collect input data for beamforming, with the objective of assessing the aeroacoustic source of an automobile at its preliminary development stage. The researchers utilized operational transfer path analysis (OTPA) for source attribution and ranking. The OTPA is proficient for automotive applications, especially in evaluating the contributions of components such as the engine, tires, and suspension. Putner [23] utilized the OTPA to evaluate the influence of engine noise and vibration on interior acoustics. This study employed 44 data channels over a duration of 25 s for data collection and utilized singular value decomposition (SVD) for analysis. All the algorithms discussed above concentrate on increasing the accuracy of source ranking. A large number of microphones are used for data collection, which consumes time and increases the cost.

Several researchers have used sound quality analysis to assess and rank the sound sources [24,25,26,27,28]. The sound quality assessments are predominantly utilized for assessing complete equipment rather than individual sources within the equipment, and use juries or subjects for evaluation. The methods discussed in the literature are well suited for free-field environments, but they require significant modification when used in a reverberant environment. New research initiatives have concentrated on reducing the testing expenses, which has led to various new strategies for reverberant environments [29,30]. Innovative algorithms employing methodologies such as minimum variance (MV), multiple signal classification (MUSIC), cross-correlation (CC), and cross-power spectrum phase analysis (CSP) were developed by [31,32,33]. Originally designed for reverberation-free conditions, the researchers adapted these methods to apply in reverberant environments. The cross-correlation coefficient played a major role in the methods discussed in the literature. Cho et al. [34] considered a source as the loudest if the correlation value was high and ranked the remaining sources in descending order. These methods are efficient but use a large amount of data.

Currently, supervised machine learning models are utilized to identify audio events and rank them. The machine learning models require a significant amount of ground truth data to train the system before being applied to source ranking and localization. Opochinsky et al. [35] used a restricted quantity of labeled samples to identify the sound sources in a reverberant environment using a deep learning methodology. Similar methods have been utilized for source ranking [36,37,38,39,40,41,42,43,44]. Some researchers have employed ad hoc machine learning models for sound source localization and ranking [45,46,47,48,49,50,51,52]. These models utilize graphics to detect sources and the key components of sound-activated robotic systems. The methods discussed so far in the literature provide an extensive examination of sound sources in equipment, although they are expensive and computationally intensive.

The following list provides a summary in relation to the currently employed methodologies:

- All source localization and ranking methods use large microphone arrays to cover the entire face of the equipment in the study, irrespective of the testing environment.
- The experiments generate a large volume of data.
- The large volume of data necessitates an extended processing duration.
- The methods used to assess the noise components quickly during the initial phases of product development are inadequate.
- All the above methods are expensive and time-consuming.

The methodologies currently in practice can be classified as traditional and machine learning (ML)-based. Both these methodologies use a large amount of data for source identification. So, the data are collected either with a few sensors over an extended period or with many sensors over a shorter period. Setting up sensors to collect the data is time-consuming. Most of these methodologies are based on STFT and require at least a minimum of nine microphones to resolve the sources. These methodologies also require methods like near-field acoustic holography (NAH), beam-forming, or the use of algorithms like MUSIC, GCC-PHAT, etc., to resolve the sound sources. The STFT-based methodologies require a minimum of two samples to resolve the frequencies. Considering the number of microphones and the use of two samples per microphone, the volume of recorded data is high. Processing this high-volume of data using the above-mentioned algorithms and methods contributes significantly to the overall time of the ranking studies. However, applications such as engine testing during development require a specialized method to identify and rank the sources very quickly. So, a requirement for such a methodology is that it works with less sensor data, processes the signal quickly, and operates in a reverberant environment. The proposed methodology is designed such that the sound signal of the engine can be collected using a single microphone in an engine test cell during its performance test. Though the DMD-based algorithm requires more memory and time to resolve the frequencies, it uses only a single microphone to collect a few seconds of data. This simple setup reduces the overall time to resolve and rank the sources in a reverberant environment. The following Section 2 describes the methodology. Section 2.1 addresses data acquisition and DMD. The final section discusses the results derived from the experiment and the data processing of the signal, followed by the conclusions.

## 2. Materials and Methods

**Figure 2:**
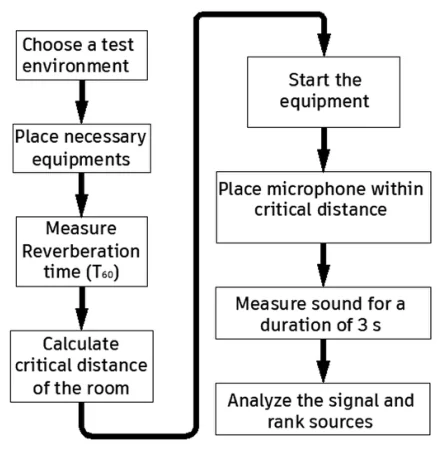
Methodology for ranking sound sources.

A signal processing technique that yields positive results with a low signal-to-noise ratio enables researchers to optimize the number of microphones and utilize any environment for data acquisition [53]. This work characterizes the sound field, systematically gathers data, and employs an efficient algorithm to rank the sources. The methodology that is proposed to attain a rapid ranking is shown in Figure 2.

**Figure 3:**
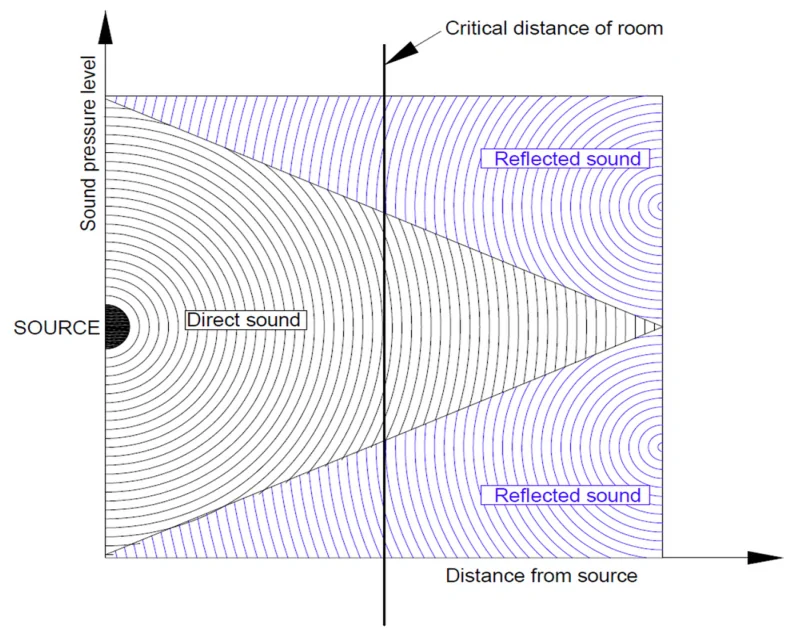
Illustration of critical distance.

Research conducted by Aida et al. [54] and Larsen et al. [55] demonstrated that at a critical distance, the SPL of the direct sound and reflected sound of a source equal each other, as illustrated in Figure 3. The critical distance affects the reception of direct sound from the source and depends on the room’s reverberation time ($T_{60}$). $T_{60}$ denotes the time interval necessary for the sound level in a room to decrease by 60 dB after its emission from a sound source [56]. Schroeder [57] devised a method to compute $T_{60}$ from $T_{35}$. This is particularly advantageous when the ambient sound pressure level (SPL) is high and achieving a 60 dB reduction in SPL is impractical. The process starts with the measurement of the room reverberation time and then subsequently involves determining the critical distance. The critical distance is defined in Equation (1). Using the reverberation time $\left(T_{60}\right)$, the volume of the room (Vr), and an omni-directional sound source assumption (Q=1), the critical distance is calculated using Equation (1).

(1) $$d_{cr}\approx 0.057\sqrt{\frac{V_{r}Q}{T_{60}}}$$

The amplitude of a sound wave diminishes as it propagates farther from the source [58,59]. Schmid et al. [60] used a decay factor to represent the decay of the signal when decomposed by DMD. In this study, DMD’s decay factor represents the amplitude decay of the sound wave. The decay factor is defined in Equation (2). The decay factor decreases exponentially for a wave leaving away from the source, representing the decrease in the sound pressure level. If the decay factor stagnates or increases, the sound wave is influenced by reverberation or other disturbances, which can lead to an increase in sound pressure levels.

The Fast Fourier Transform (FFT) is a proven algorithm utilized by researchers to convert a signal from the time domain to the frequency domain, thereby revealing the sound sources present in the system. The FFT provides only the frequency contents, leaving aside the temporal contents of the signal. So, the Short-time Fourier Transform (STFT) was used to include the temporal content. DMD by nature captures the temporal content. The proposed methodology utilizes the decay factor derived from DMD to separate and rank the sound sources.

(2) $$Decay\;factor\;\left(DR\right)=\frac{Change\;in\;amplitude}{Duration\;between\;two\;timesteps}$$

### 2.1. Dynamic Mode Decomposition

DMD exhibits a variant of Koopman theory, employing experimental data to forecast patterns derived from data snapshots. At its first implementation by Schmid [60] in 2008, DMD enabled solution of a complex fluid mechanics problem. Researchers have employed DMD for numerous engineering applications [61,62,63,64,65,66,67,68,69,70,71]. DMD analyzes both spatial and temporal data to forecast frequencies, amplitudes, and their dynamics. Singular value decomposition (SVD) serves as the foundation of dynamic mode decomposition (DMD) for signal de-noising while retaining pertinent information for subsequent processing [72,73]. The current work primarily uses DMD modes and variations in their amplitude to evaluate the sound sources [74].

Time-series data contain extensive information regarding their origin. DMD derives additional insights from the time-series data [75]. The DMD forecasts dominating modes in an auditory signal by altering the snapshot configuration. The DMD formulation is presented in Equation (3).

(3) $$P_{i=1}^{j=0}=p_{1}^{0},p_{2}^{0},p_{3}^{0},...,p_{N-1}^{0}P_{i=1}^{j=1}=\left\{p_{1}^{1},p_{2}^{1},p_{3}^{1},\ldots ,p_{N}^{1}\right\},$$

Both $P_{i}^{j}$ and $P_{i}^{j+1}$ represent a sequential set of data snapshots. Indices *i* and *j* represent the data within a snapshot and number of snapshots, respectively. *N* represent the total quantity of data within the snapshot. *M* represent the total number of snapshots. Both $P_{i}^{j}$ and $P_{i}^{j+1}$ establish an optimal linear correlation matrix *A*, as specified in Equation (4).

(4) $$P_{i}^{j+1}\approx AP_{i}^{j},$$

can be reformulated as indicated in Equation (5),

(5) $$A=P_{i}^{j+1}P_{i}^{j*}$$

The matrix $P_{i}^{j*}$ is the pseudo-inverse of $P_{i}^{j}$. The matrix *A* consists of the dynamic parameters as eigenvalues and eigenvectors of the process. As it represents the entirety of that moment, its size becomes excessively large for computers to manage. DMD addresses this issue by decomposing the matrix *A* just when significant change occurs between two successive data points in the results. The aforementioned methodology denotes the singular value decomposition (SVD). The singular value decomposition of $P_{1}^{0}$ and Equation (6) delineates the procedure.

(6) $$P_{1}^{0}\approx R_{1}\Sigma R_{2}$$

where $R_{1}$ is of size N×r, Σ is of size r×r, and $R_{2}$ is of size r×M. Substituting Equation (6) into Equation (5) yields Equation (7).

(7) $$A=P_{i}^{j+1}R_{2}\Sigma^{-1}R_{1}^{*}$$

The matrix *A* has dimensions of N×M and necessitates size reduction for enhanced signal processing by a computer. The rank *r*, which limits the quantity of dominant modes between snapshots, aids in minimizing the dimensions of the matrix *A*. The rank reduction procedure is illustrated in Equation (8).

(8) $$\tilde{A}=R_{1}^{*}AR_{1}=R_{1}^{*}P_{i}^{j+1}R_{2}\Sigma^{-1}$$

The matrix $\tilde{A}$ encompasses the DMD modes and their corresponding eigenvalues, as illustrated in Equation (9).

(9) $$\tilde{A}W=W\Lambda$$

where *W* denotes the modes and Λ signifies the appropriate eigenvalues, as seen in Equation (10).

(10) $$\varphi =P_{i}^{j+1}R_{2}\Sigma^{-1}W$$

The columns of the φ denote the precise DMD modes of *A*. The aforementioned procedure decomposes the signal into DMD modes and their associated eigenvalues, which represent frequency of occurrence and rate of change. The DMD decomposes any time series signals into modes, necessitating conversion of the data to accurately reflect the associated sound signal parameters, such as amplitude and frequency. The DMD modes denote amplitude, while the eigenvalues indicate frequency and increasing rate of occurrences.

(11) $$\Omega =\frac{\log(\Lambda )}{\delta t\cdot 2\cdot \pi }$$

Equation (11) computes the continuous rate of change in modes.

(12) Λ=g+if

where Λ and Ω represent the eigen value and frequency of a mode, respectively. The values *g* and *f* denote the growth rate and the frequency of oscillation in Equation (12). The rates of change are significant as they indicate the variation in each mode and teach us about the behavior of the sound source signal.

### 2.2. Comparison Between Dynamic Mode Decomposition vs. Short-Time Fourier Transform

Short-time Fourier Transform (STFT) is a default method for resolving the frequencies of sound signals. A comparison between DMD and STFT is presented in this section. STFT resolves the signals using fixed sinusoidal waves. DMD uses a linear operator to best fit the measured data. STFT provides frequency and amplitude values of a signal. Another property of STFT is identification of the occurrence of a frequency in the signal. Apart from the frequency and amplitude, DMD is able to find the decay factor of each frequency. The decay factor provides important information: the nature of the amplitude decay of a mode. This information is essential to identify the sound source. The fundamental differences between STFT and DMD are shown in Table 1. The comparison given in Table 2 shows that DMD is consistent over a wide range of SNR values as reported by the authors [76].

**Figure 4:**
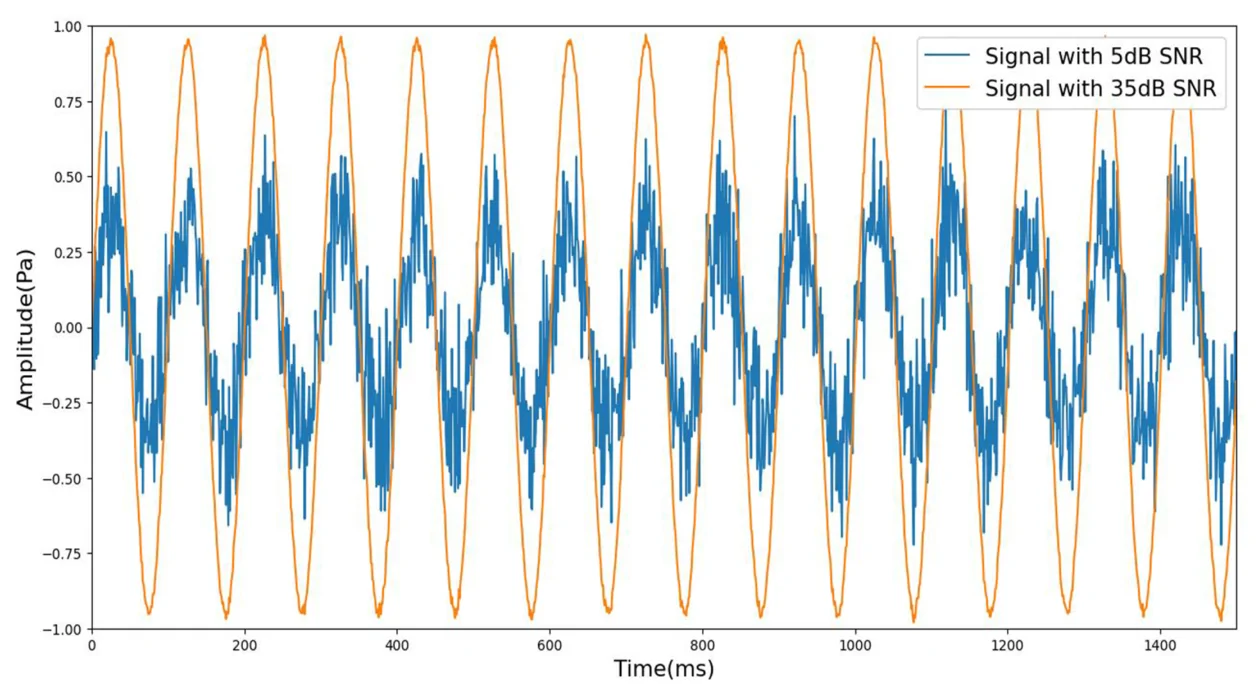
Ground truth signal with 5 dB and 35 dB SNR.

Figure 4 shows the signals used for the comparison study. A computer with 8 GB RAM, an i5 intel core processor, and 4GB graphics memory is used. A Python program is developed for DMD as well as the STFT calculations. The Python version used is 3.12. DMD uses fewer samples to arrive at the results. As the number of samples reduces, the DMD maintains a reasonable accuracy over STFT. Table 3 shows the cross-correlation values calculated by the DMD and the STFT. Both STFT and DMD are compared for their performance in terms of memory usage and execution time, as shown in Table 4 and Table 5. Throughout this study, STFT uses the “Hanning window.” Though the memory usage and execution time by DMD are higher than STFT, DMD maintains the accuracy level irrespective of the SNR of the signal. Considering this, all further sound signal processing in this study is carried out using DMD with a snapshot size of 24,000.

In the preceding two sections, two principal elements influence the source ranking: the sound field estimate and signal processing. The remainder of the paper aims to rank the known sources of an engine—a multi-sound source system—in a reverberant environment using a single microphone for signal capture, minimizing time and effort. The next section outlines the experimental setup used in this study.

### 2.3. Experimental Setup

The experiments are designed to prove the effectiveness of the methodology in two testing environments. The first environment is a silent room, and the second is an engine test cell. The methodology starts with characterization of the testing environment. At first, the silent room is considered. It has a sound field with less reverberation and external disturbances. In this room, only pure-tone signals are used. The reverberation time $\left(T_{60}\right)$ determines the sound field of a closed environment, and the balloon experiment described in [77,78,79] is conducted to measure the same.

Three experiments were conducted in the silent room. The first experiment assesses the critical distance of the room, the second calculates the decay factor of the sound waves, and the third ranks the sources. All these experiments use speakers as signal generators [80].

**Figure 5:**
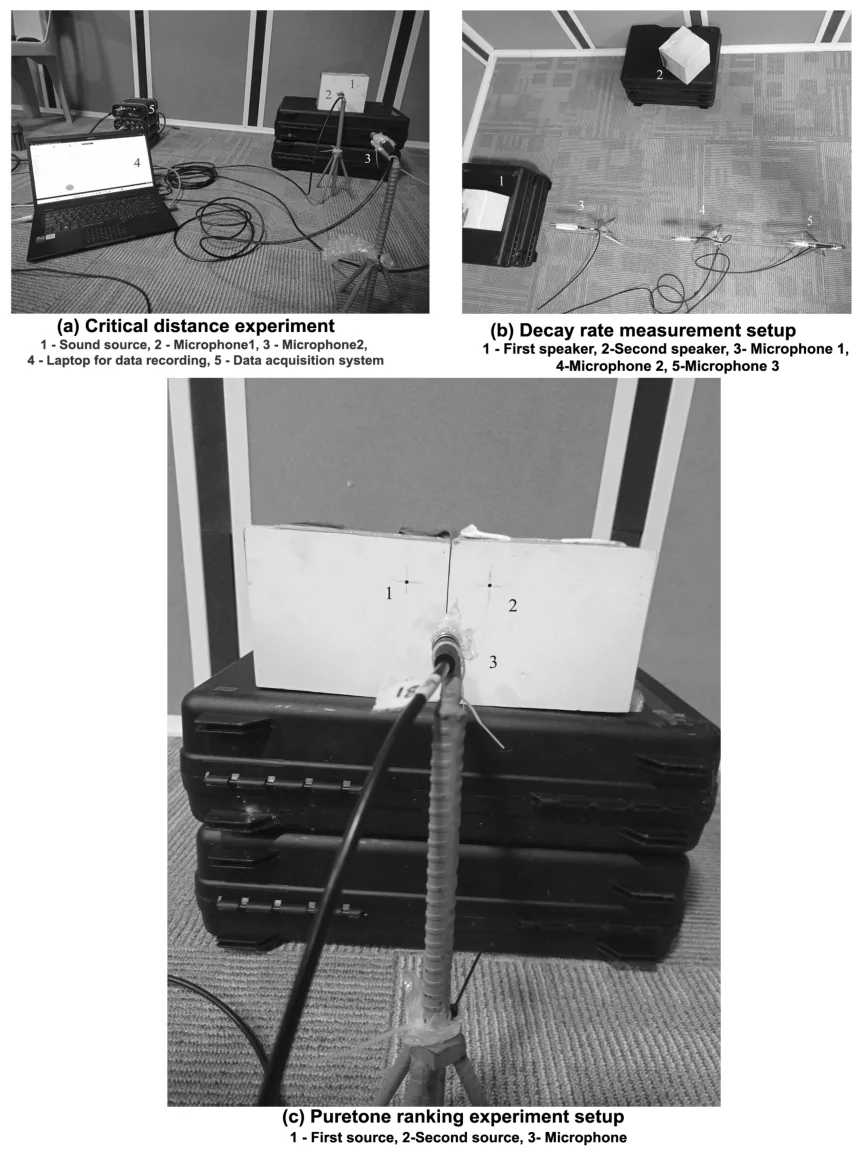
Experiment setups for ground truth data collection.

In the first experiment, a speaker producing a pure-tone sound is used as a source. Two microphones are placed at 5 cm and 55 cm from the source to collect the data using a data acquisition system (DAQ), as shown in Figure 5a. The microphones are moved subsequently to collect data at various distances. Table 6 shows the specifications of all sensors used in this paper.

In the second experiment, two speakers and three microphones are used. The first speaker, shown in Figure 5b, represents the main source, and the second speaker represents a reflected sound. The first and second speakers both play the same frequency signal with a different amplitude. The second speaker plays at 40% of the first speaker’s amplitude in order to simulate a reverberant environment. The microphones are positioned so that the first one collects a maximum of direct sound, the second one collects the direct and simulated reverberation sound, and the third one collects both signals from outside the critical distance from the first speaker.

The third experiment uses a set of speakers as a multi-sound source system. Both the speakers play pure-tone signals of two different frequencies and amplitude. Figure 5c shows the experimental setup. Considering the critical distance of the silent room, the microphone is placed at 5 cm from the source.

**Figure 6:**
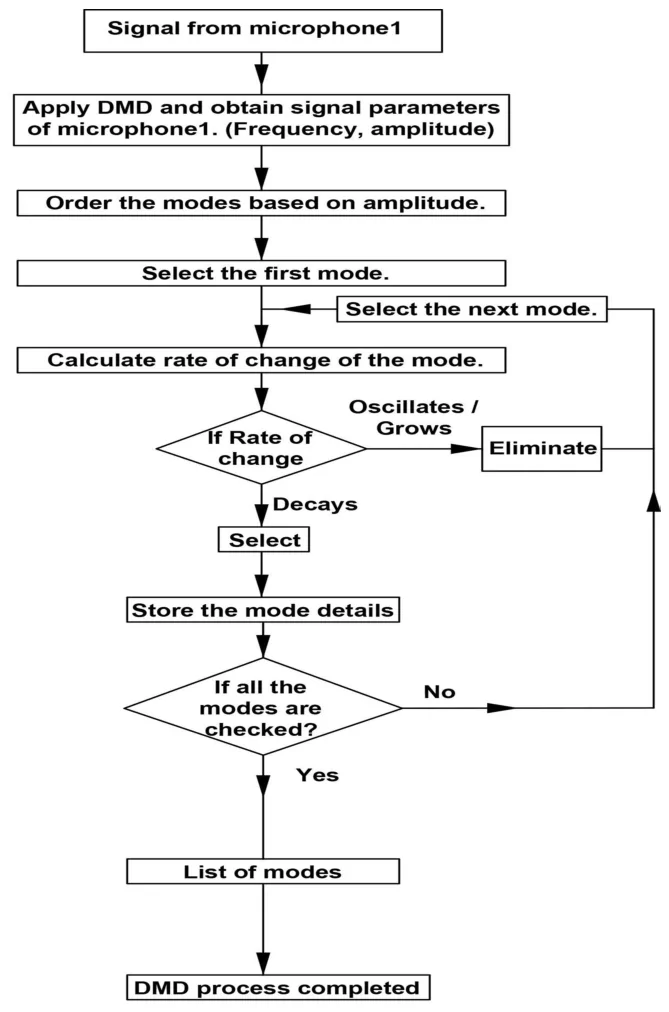
Algorithm for quick ranking of sources.

The three experiments discussed above use a silent room and pure-tone signals to assess the effectiveness of the proposed methodology in ranking sources in a multi-sound source system. The next section ranks the sources present in a multi-sound source system, and the methodology is shown in Figure 6.

The experiment discussed further applies the methodology to a real-life problem: separation and ranking of the sources in an internal combustion (IC) engine. The proposed methodology ensures ranking of all the sources in an IC engine as it represents a multi-sound source system and fits perfectly to test the methodology as previously discussed [81,82,83,84]. The experiment is conducted in a regular engine test bed with acoustic treatment on all sides except the floor. The dimensions of the engine test bed are 6 m width, 7 m length, and 4 m height. The engine test bed is chosen because the proposed methodology is designed for early detection and ranking of sound sources during the development phase.

**Figure 7:**
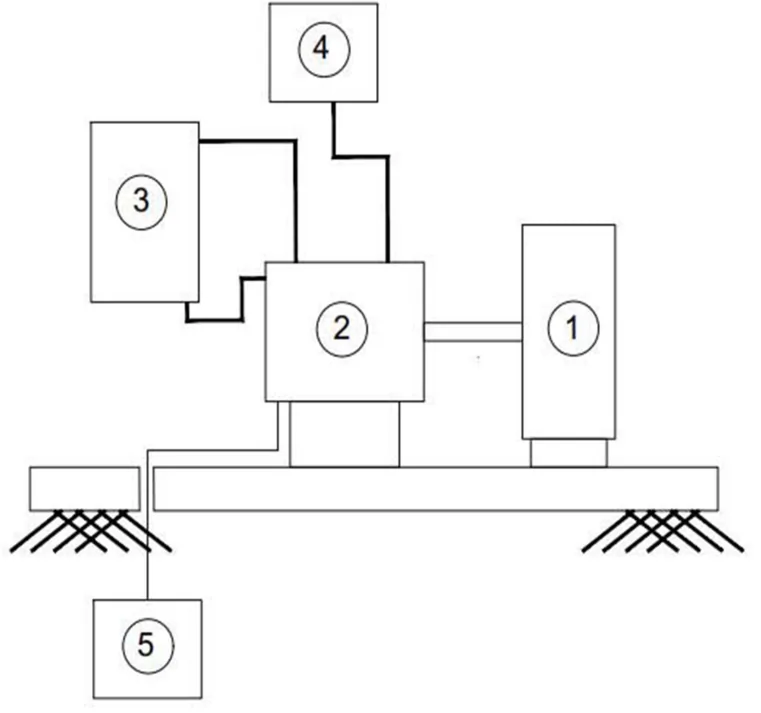
The experimental setup for IC engine testing. 1—Eddy current dynamometer; 2—IC engine; 3—Coolant conditioning system; 4—Fuel conditioning system; 5—Exhaust system.

**Figure 8:**
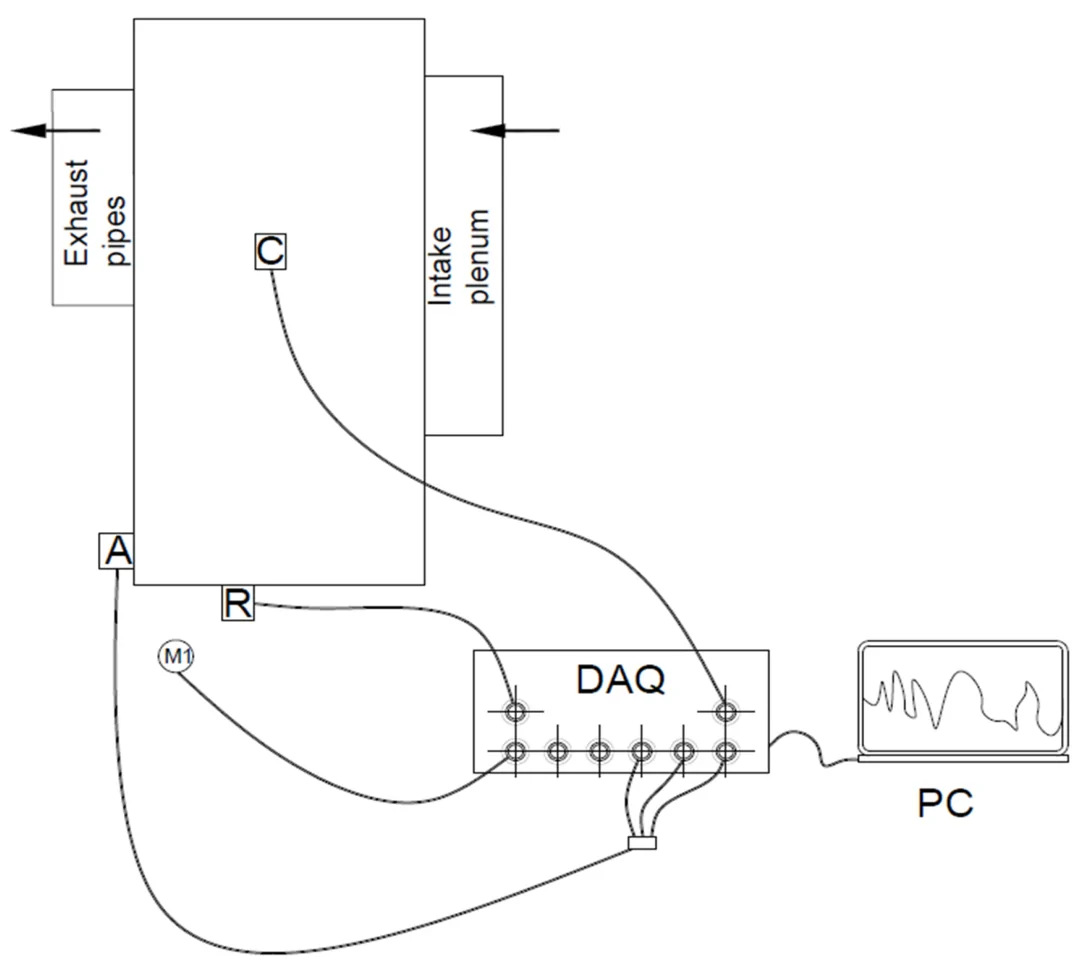
Sensor arrangement in the experimental setup. A—Accelerometer; M1—Microphones; R—Speed sensor; C—Cylinder pressure sensor.

The experiment setup comprises an 80 kW eddy current dynamometer and necessary accessories like a coolant conditioning system and fuel conditioning system for the engine testing. Figure 7 shows the experimental setup. Due to a non-disclosure agreement (NDA), the setup schematic only is shown in Figure 8. The experiment setup includes a microphone and an RPM sensor as primary sensors. Auxiliary sensors, an accelerometer, and a cylinder pressure sensor are included to measure and correlate the engine vibrations with airborne noise and structure-borne noise. During data collection, the engine is operated at 2000 and 4250 rpm. The microphone is placed at 10 cm from the outermost part of the engine, which falls within the critical distance of the room.

The next section discusses the results obtained from the above-discussed experiments.

## 3. Results

**Figure 9:**
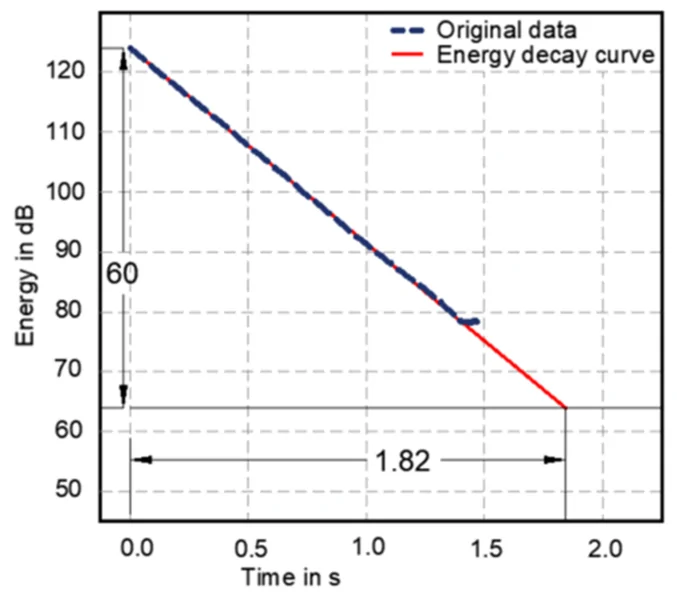
Sound energy drop during $T_{60}$ measurement in the silent room.

The methodology starts with evaluation of the testing environment. The first three experiments using pure-tone signals are conducted in a silent room. The reverberation time $\left(T_{60}\right)$ of the silent room is 1.82 s as extrapolated from Figure 9. The critical distance of the silent room is 43.7 cm.

**Figure 10:**
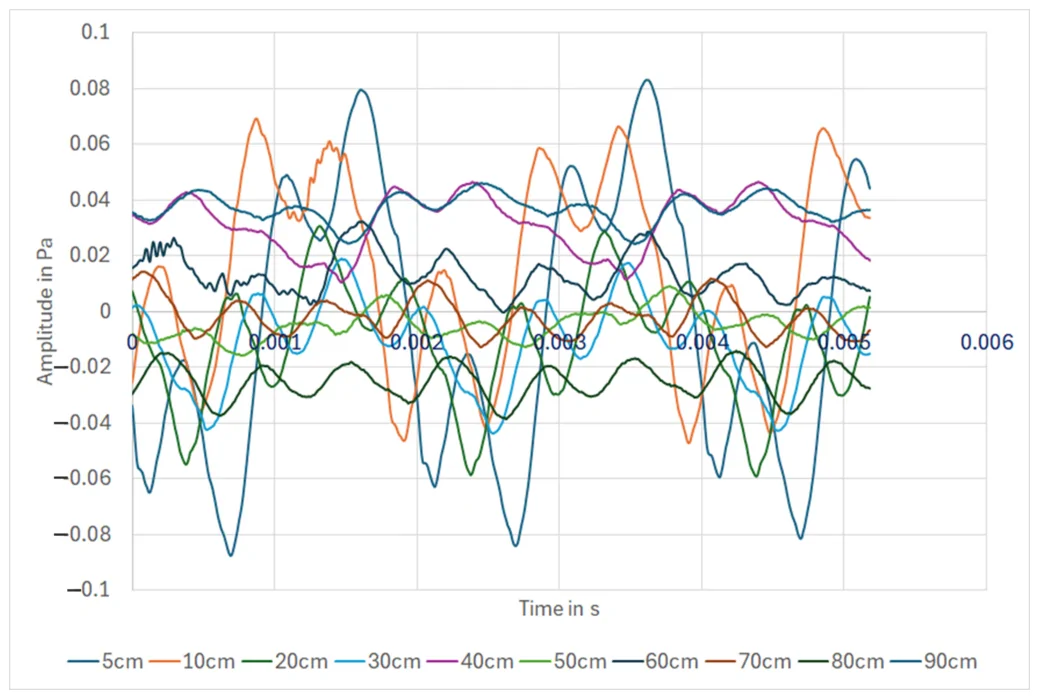
Sound signal recorded at various distances from the source.

**Figure 11:**
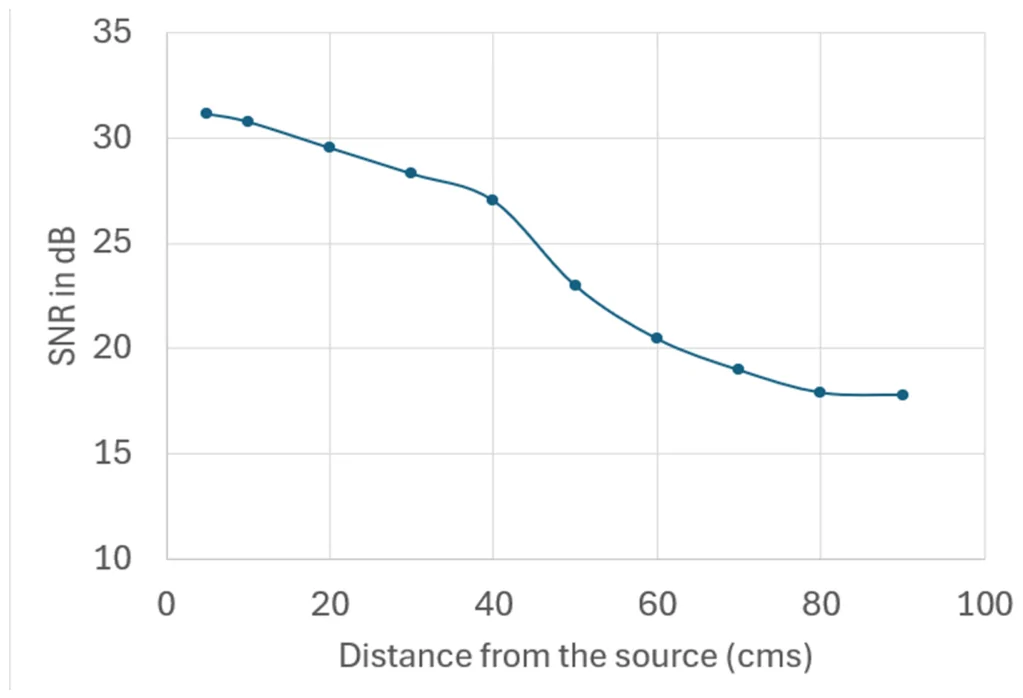
SNR at various distances from the source.

Figure 10 shows the signals recorded at various positions from the source. As seen in Figure 11, the SNR of the signal deteriorates sharply after the critical distance and reflects the increase in the reverberation noise level in the recorded sound signal.

Table 7 shows the amplitude and signal-to-noise ratio (SNR) of the recorded sound signals at various locations of the microphones. This shows that signals recorded within the critical distance have good SNR. The next step checks the correlation between the decay factor and the critical distance.

**Figure 12:**
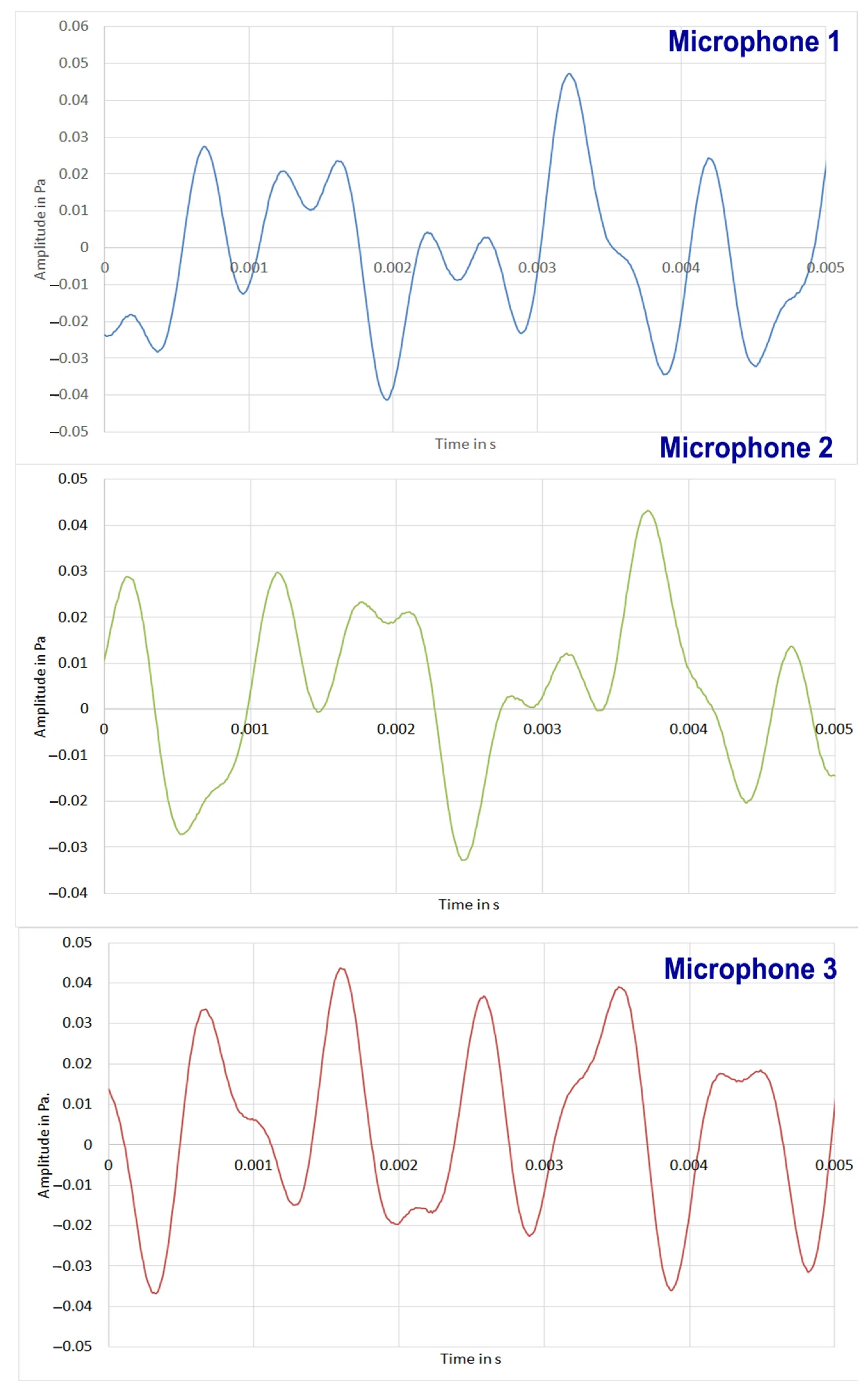
Sound signals recorded in the decay rate experiment.

**Figure 13:**
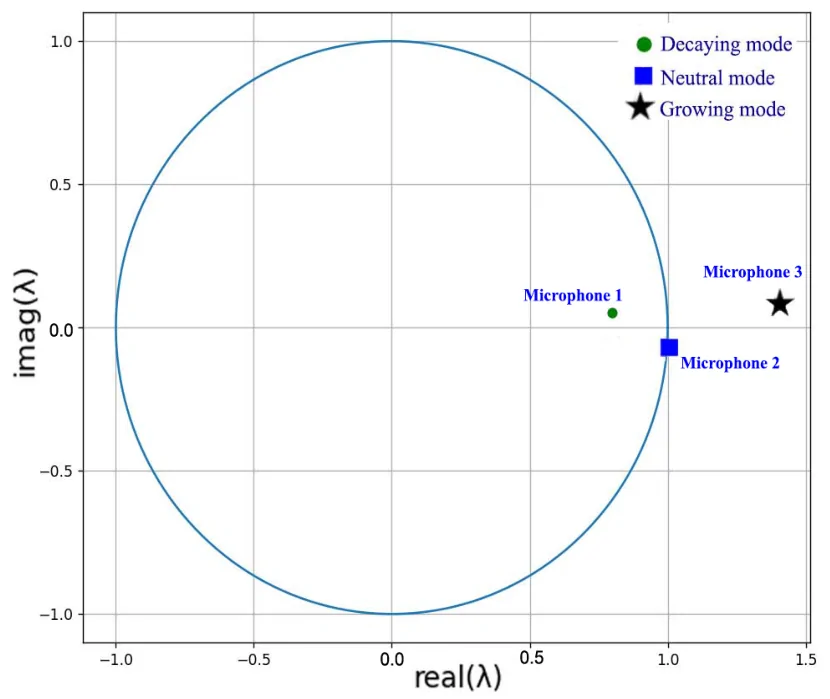
Decay factor plot for pure-tone signals from the second experiment.

Figure 12 shows the signals recorded in the second experiment. The DMD separates the modes of the signal along with their decay factor, as shown in Table 8. If the decay factor value is negative, the mode is decaying; if it is unity, it is neutral/oscillating; and if it is positive, the modes are growing. Figure 13 shows a plot of the decay factor of the microphone signals. The results show that microphone 1 captures more direct signal from the source, and as the microphone’s position is moved away from the source, the signal is mixed with more reverberation sound.

**Figure 14:**
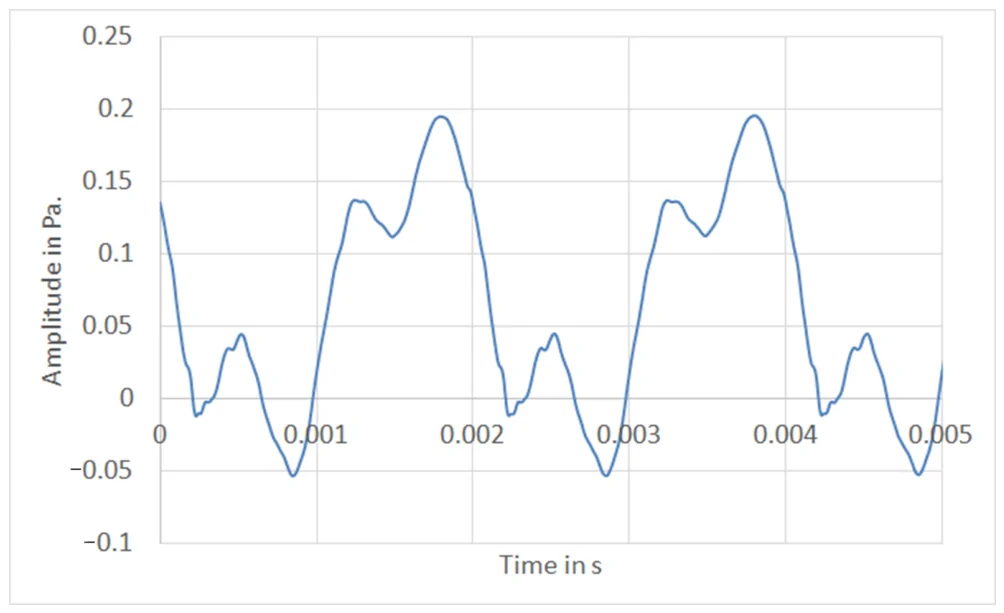
Signal recorded during ranking experiment.

**Figure 15:**
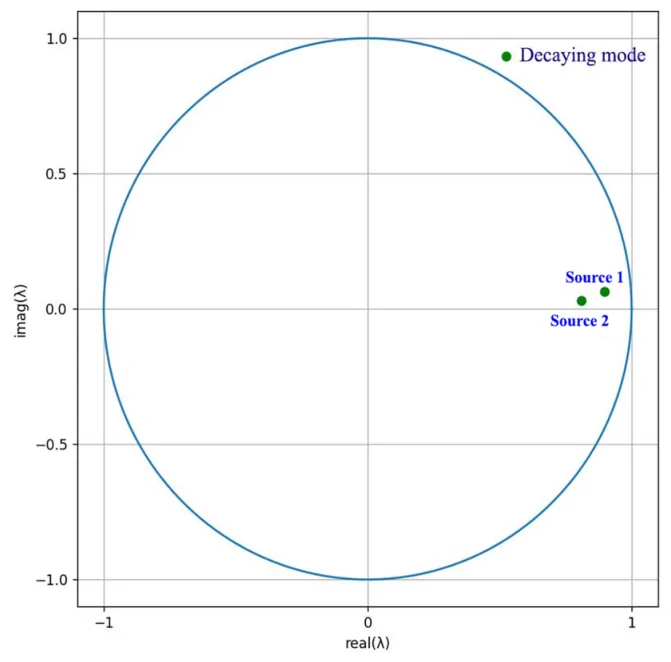
Ranking of pure-tone sources from a multi-source sound system used in the third experiment.

The sample sound signal shown in Figure 14 is recorded for 3 s in the third experiment. Table 9 and Figure 15 show the DMD values of the sources of pure-tone multi-sound source system. This shows that DMD is able to separate and rank the sources from a multi-sound source using a single microphone placed within the critical distance.

**Figure 16:**
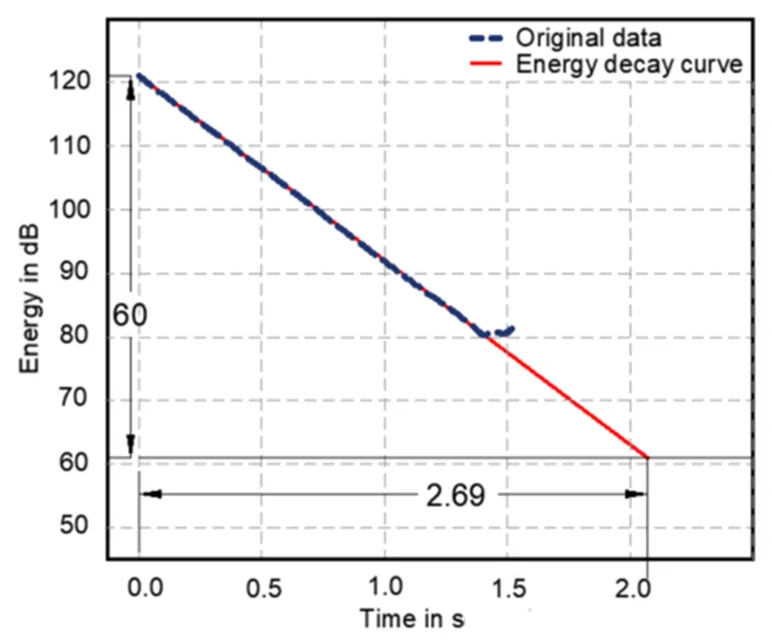
Sound energy drop during $T_{60}$ measurement in the engine test cell.

The further discussion of the results here focuses on the outcomes of the application of the methodology in a real-life experiment. As discussed, an engine test cell is considered as the test environment. The critical distance is calculated from the reverberation time $\left(T_{60}\right)$ of the engine test cell, which is 2.69 s as derived from the plot in Figure 16. The sound signals are also collected at 90 cm from the engine in order to show the difference between the two measurement positions. The collected data are analyzed using DMD.

The modes decomposed by DMD are compared against three parameters: analytical values calculated from the fundamental equation, the frequency of vibration, and the frequency of combustion pressure of the engine.

The analytical equation governing the firing frequency of a three-cylinder four-stroke engine is given in Equation (13).

(13) $$F_{f}=\frac{2N_{e}N_{cyl}}{60N_{s}}$$

where $F_{f}$—Firing frequency of the engine; $N_{e}$—Engine rpm; $N_{cyl}$—No. of cylinders; $N_{s}$—No. of strokes.

The fundamental relationships given in Table 10 are used to calculate the frequencies of camshaft rotation, oil pump rotation, coolant pump rotation, and other source frequencies.

The noise and vibration signals of an engine are a combination of different frequencies and amplitudes arising from compression and combustion. The non-dimensional DMD modes are correlated with engine frequencies for ranking. The accelerometer and the cylinder pressure sensors collect the vibration and combustion data, respectively. The additional parameters are collected only to ascertain the mode’s correlation with the frequencies of the engine vibration and combustion pressure. The structure-borne noise signals are obtained by comparing the frequencies of sound and vibration of the engine. Similarly, airborne noise signals are obtained from the frequencies of the sound signals and the combustion pressure signals.

**Figure 17:**
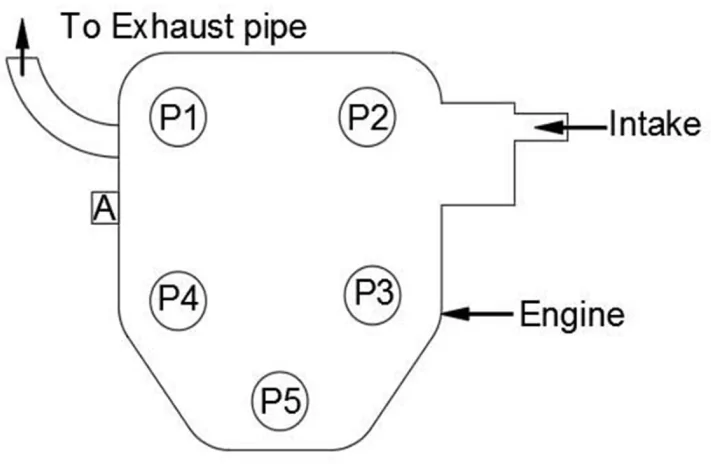
Microphone positions on the fan side of the engine. A—Tri-axial accelerometer; P1–P5—Microphone positions.

The data collection is started after switching on the engine and maintaining the speed at 2000 rpm. Along with the microphone, the auxiliary sensors also collect the data. The microphone is placed at position 1 at first and subsequently moved to other positions, as shown in Figure 17. At position 1, the microphone data are collected at 10 cm and 90 cm from the source surface. The same procedure is repeated for the engine speed of 4250 rpm.

**Figure 18:**
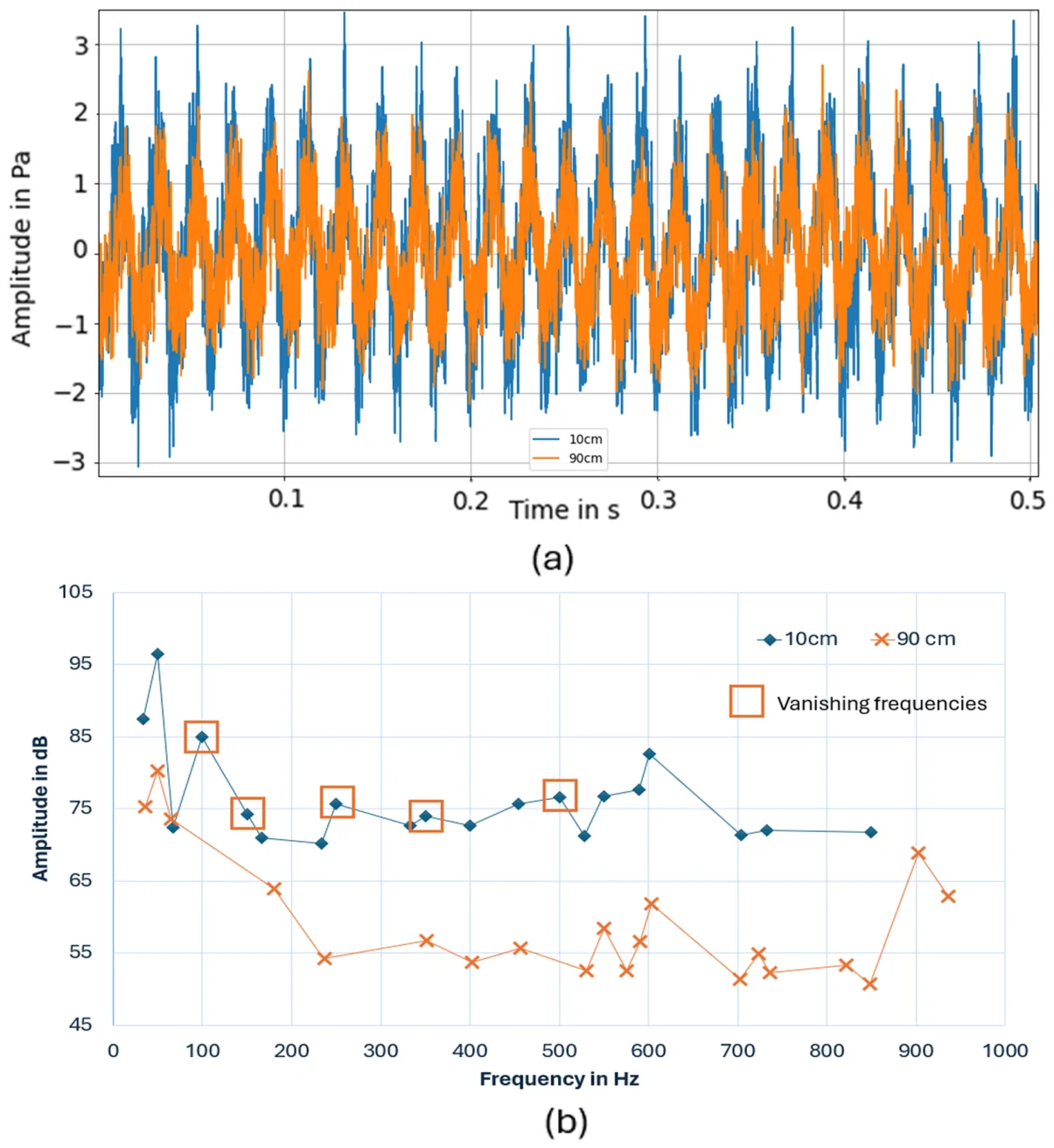
Mircophone signal data at a distance of 10 cm and 90 cm from the engine: Position 1 and engine speed of 2000 rpm. (**a**) Sound signals; (**b**) Frequency graph.

Figure 18a shows the signals recorded at position 1. DMD decomposes these sound signals to calculate the frequency components present in them. Figure 18b shows the vanishing nature of certain frequencies beyond the critical distance of the room, as discussed by Aida et al. [54] and Larsen et al. [55]. This also justifies the data collection within the critical distance from the sound source.

### 3.1. Fundamental Frequencies vs. Sound Signal Frequencies

In Table 11 and Table 12, the frequencies based on the sub-system speeds and the fundamental firing frequencies of the engine are shown along with the DMD mode frequencies at 10 cm and 90 cm distance from the engine. These tables compare the frequencies calculated from the fundamental empirical equations and the frequencies decomposed by DMD.

### 3.2. Structure-Borne Noise Separation

**Figure 19:**
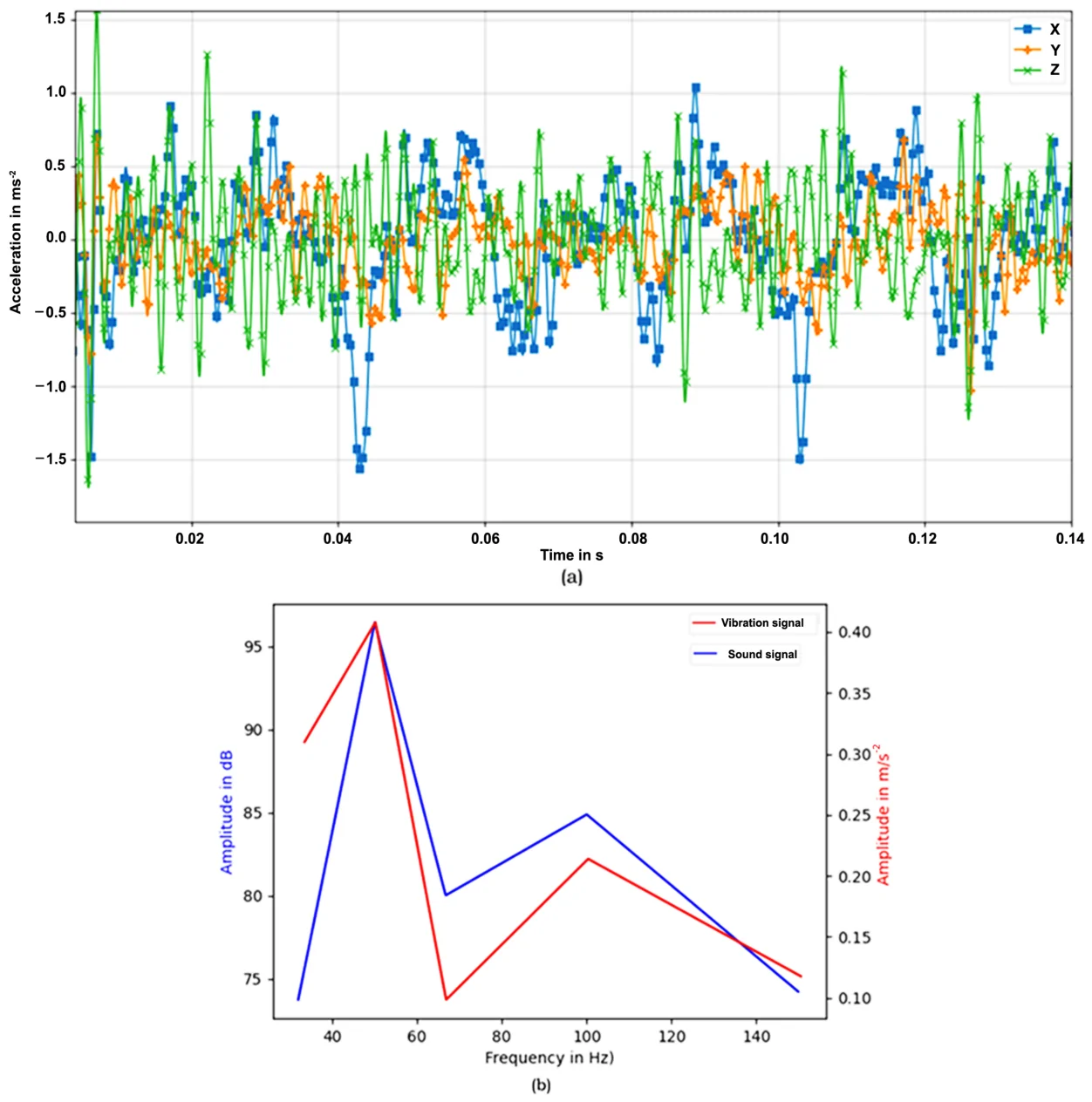
(**a**) Vibration signals recorded using a tri-axial accelerometer mounted on the engine; (**b**) Frequency vs. amplitude graph of the vibration signal and the sound signal measured at 10 cm.

The vibration of the structure is the basis for the structure-borne noises. Figure 19a shows the vibration signals collected. After discretization of both the vibration and sound signals using DMD, it is found that some of the frequencies obtained from sound signals and vibration signal match each other. This shows that the frequencies separated by DMD contain the structure borne noise signals as well.

Figure 19b shows that the DMD could identify the structure-borne noise of an engine and its match with the fundamental frequencies of the engine. Using the above, it is evident that the frequencies of the structure-borne noise from the sound source match the calculated fundamental frequencies; this undermines the need for an accelerometer for sound source localization in an engine.

### 3.3. Air-Borne Noise Separation

**Figure 20:**
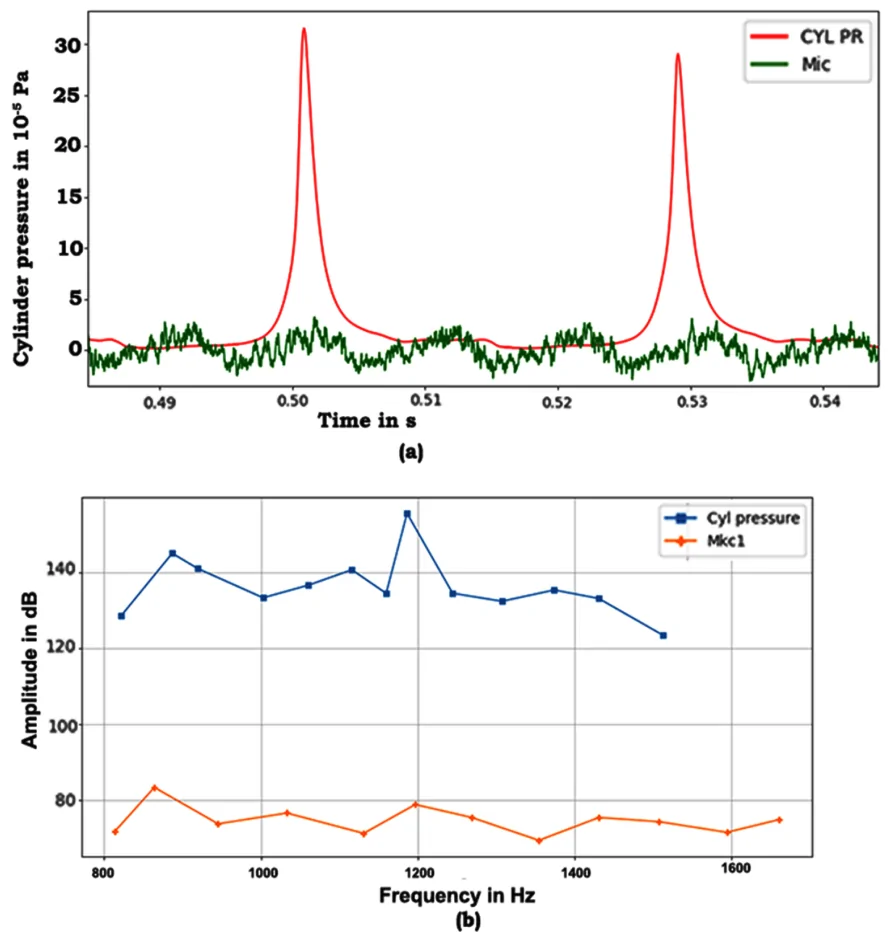
(**a**) Cylinder pressure signal vs. sound signal; (**b**) Frequency vs. amplitude graph of the cylinder pressure and sound signals recorded at 10 cm.

The major source of air-borne noise is the combustion of fuel inside the cylinder [85,86,87]. The cylinder pressure sensor is connected to the first cylinder of the three-cylinder engine. The cylinder pressure signal is shown in Figure 20a. A good correlation is observed between the cylinder pressure and the sound signal, as given in Figure 20b. It is also understood that DMD could separate the combustion-based signals from the microphone-recorded signals. In general, a separate sensor is required to record the combustion-related pressure. As DMD is able to identify the combustion of fuel inside the cylinder from the recorded sound signal, the need for a separate combustion pressure sensor is undermined.

### 3.4. Sound Source Ranking and Source Signal Verification

The rank of the DMD defines the number of modes to be considered for further processing. The rank of the DMD is essential for reducing the computation required for decomposition of the signal. There are various methods used for selecting a rank value. The decision is based on either the physics involved or the percentage of error. In this study, the physics of the problem is used to define the rank. The sound generated by an automobile is governed by ISO 5130 [88] standards. Based on these standards, a sound pressure level (SPL) of 70 dB is considered.

**Figure 21:**
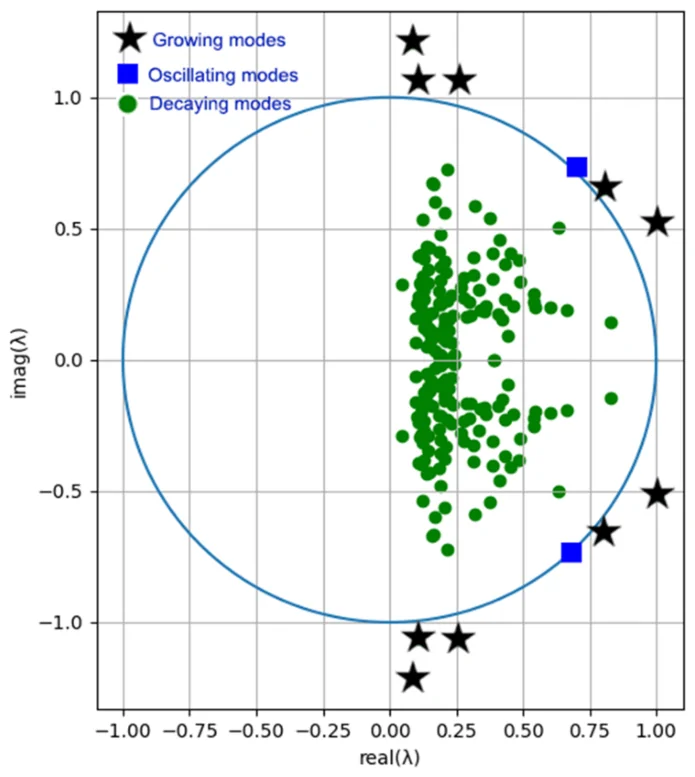
DMD modes at position 1 (P1) in a unitary circle.

The selection based on the rank of DMD contains all types of modes. In order to identify that the sound is from one of the sources of an engine, further study is required. The modes with amplitudes above 70 dB are given in Table 13. Further, the decay factor obtained from the DMD is used to identify the origin of sources. The modes of the DMD can decay, oscillate, or grow as determined by the rate of change represented by the decay factor. The rate of change of modes is normalized and plotted as a unitary circle graph. Figure 21 shows the modes obtained at position P_1_. This represents the condition of each mode and helps in eliminating the unwanted modes. In the case of sound signal decomposition, the modes should decay as described in Section 2.1. So, the modes that are oscillating and growing are eliminated. These decaying modes indicate that their origin is in the engine.

**Figure 22:**
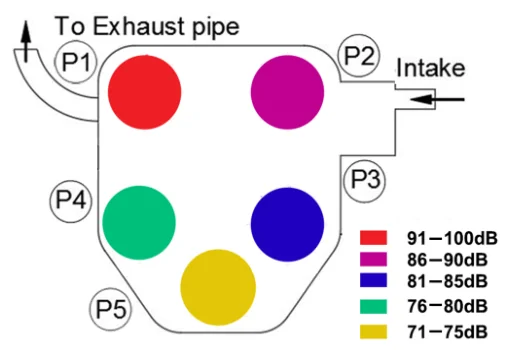
Conventional sound source localization plot.

**Figure 23:**
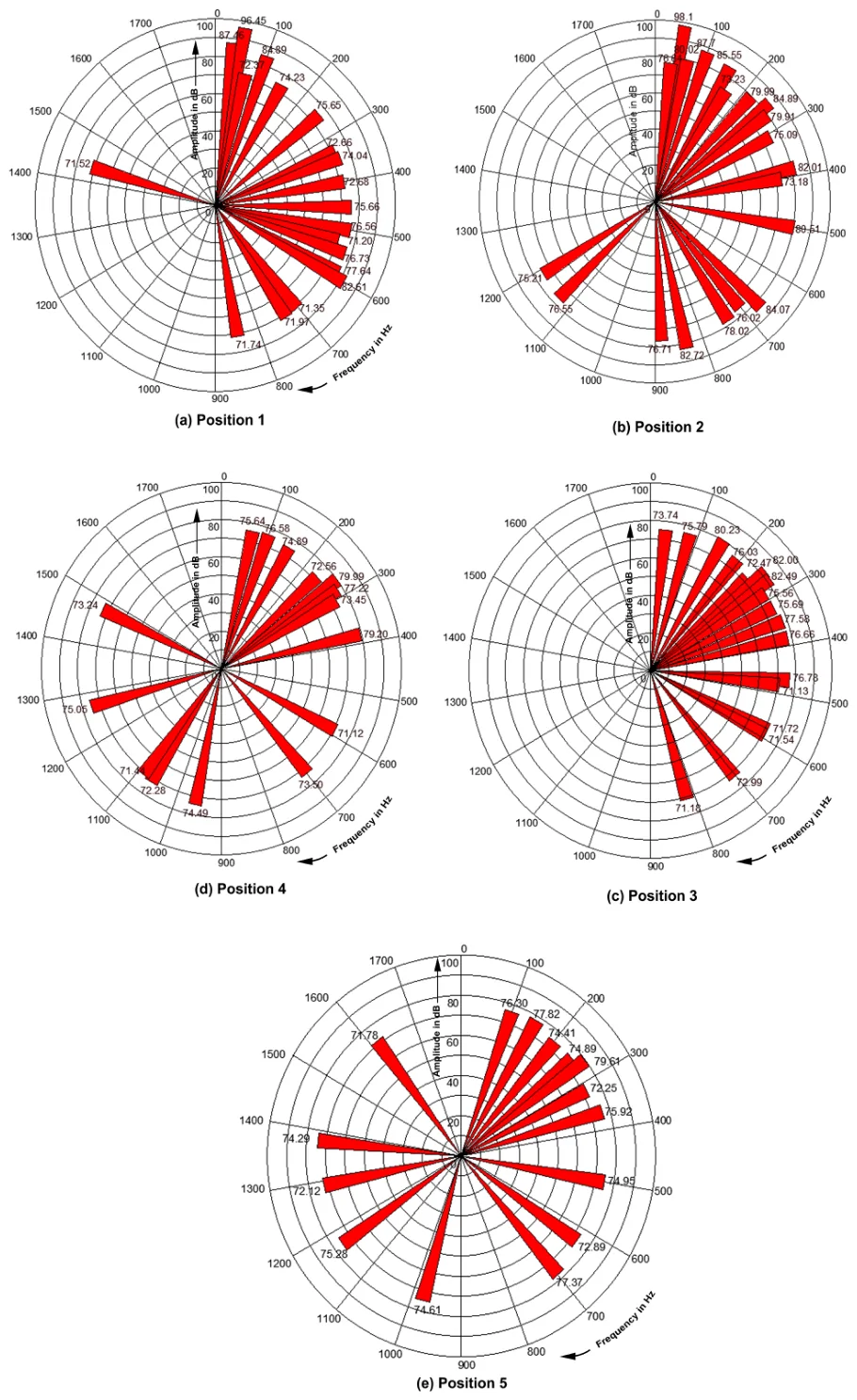
Sound frequencies measured at positions 1 to 5.

The major focus lies in the frequencies with amplitude above 70 dB as per the ranking method selected. A polar bar graph is plotted by taking the amplitude in the radial direction and the frequency in the circumferential direction. The polar bar plot at a particular position shows all the frequencies with amplitude above 70 dB. The polar bar plot is a better representation than the conventional plot shown in Figure 22. The polar plot provides a clear insight about the frequencies during the initial test phase itself. Figure 23a shows the decaying modes obtained at position P_1_.

From Figure 23a, it is understood that the mode with 50 Hz has the maximum amplitude. This figure provides a visual impression of the presence of all the dominant frequencies identified at position P_1_.

Similarly, the plots at other positions, namely, from P_2_ to P_5_, are shown in Figure 23b–e, and the corresponding values are given in Table 13. For clarity of the plots, only the decaying modes are plotted, and their values are given in the table.

Table 14 shows the frequencies identified by DMD and their relationship with the engine frequencies and the presence of them at various measurement positions.

## 4. Discussion

The results discussed above show that measurement at a position is sufficient to identify all the dominant frequencies of an engine, a multi-sound-source system. This significantly reduces the time required to identify the dominant frequencies in the initial development stage of an engine. Though a single microphone is sufficient for identifying and ranking all the sources of a multi-sound-source system, due to the reverberant nature of the environment, several more positions are used for sound signal recording. This methodology covers the entire surface of the engine. It works well for a system with known frequency parameters. In the case of a newly developed system, the auxiliary sensors discussed above need to be used for better results. The auxiliary sensors also help in resolving sources with close frequencies. As the methodology discussed in this study is specific to the ranking of the distinct sources of an engine or equipment in its prototype stage, other cases are not considered.

## 5. Conclusions

This study proposes a quick ranking methodology of sound sources in multi-sound source equipment, using a microphone in a reverberant environment. Methodologies based on the STFT and DMD are compared. Due to the reverberant environment, apart from the frequency and amplitude values, the origin of the source signal plays a role in the ranking. DMD’s inherent ability to represent the physical system helps in identifying the source signal origin. The decay factor obtained from DMD predicts the nature of the sound signal. The methodology based on STFT requires detailed data acquisition and use of additional methods to resolve the origin of the source. The DMD-based methodology is tested using multi-source pure-tone sounds as ground truth data in a controlled environment to determine its effectiveness. The same methodology is applied to an engine in a regular engine test cell to assess the effectiveness. The results match with the actual parameters of the engine. The DMD-based methodology requires far less time compared to the STFT-based methodology. The proposed sound source ranking methodology minimizes the data acquisition time required by noise and vibration engineers. The conclusions are as follows:

- Assessment of the testing environment is essential for identifying a correct signal recording position.
- The dynamic mode decomposition (DMD) algorithm is consistent over a range of SNR values of the signal. The average SNR value is 26.71 dB.
- DMD’s rate of change of the mode’s amplitude parameter, called the decay factor, is very useful in identifying the source of a signal in a reverberant environment.
- The DMD-based methodology uses less time and can be exploited for dynamic studies of sound source localization.
- Source ranking using DMD-based methodology in regular test beds proved beneficial, as it reduced the overall study time and cost and eliminated the need for an anechoic chamber.

The methodology used in this paper proved to be very effective, and can be applied to other sound source ranking studies as well.

## Figures and Tables

**Table 1:** Comparison between STFT and DMD.

Parameter∖Method	STFT	DMD
Basis function	Sinusoidal-based–predefined	Data driven and dynamic
Resolution	Affected by the size of the window selected	Affected by the sampling rate
Frequency	Yes	Yes
Amplitude	Yes	Yes
Decay factor	No	Yes
Captures physics of the problem	No	Yes

**Table 2:** Cross-correlation study between STFT vs. DMD with respect to SNR.

SNR (dB)	STFT	DMD
5	0.977	0.981
10	0.989	0.992
15	0.994	0.993
20	0.994	0.994
25	0.995	0.996
30	0.995	0.999
50	0.996	0.999
100	0.996	0.999

**Table 3:** Cross-correlation study between DMD vs. STFT with respect to the sampling duration for ground truth data and real-time data.

Number of Samples	Sampling Duration (s)	Ground Truth Data		Real-Time Data	
		STFT	DMD	STFT	DMD
24,000	0.54	0.98	0.99	0.91	0.96
12,000	0.27	0.94	0.99	0.92	0.95
6000	0.13	0.93	0.99	0.89	0.94
3000	0.06	0.24	0.99	0.22	0.92
1000	0.02	0.09	0.99	0.09	0.91

**Table 4:** Memory usage (MB) study between DMD vs. STFT with respect to the sampling duration and number of samples.

Number of Samples	Sampling Duration (s)	Ground Truth Data		Real-Time Data	
		STFT	DMD	STFT	DMD
24,000	0.54	154.704	4006.56	135.79	4612.67
12,000	0.27	152.192	1182.21	133.54	1073.43
6000	0.13	150.48	431.752	128.56	496.36
3000	0.06	149.92	244.728	126.23	456.12
1000	0.02	149.368	180.816	124.49	400.73

**Table 5:** Execution time (s) study between DMD vs. STFT with respect to the sampling duration and number of samples.

Number of Samples	Sampling Duration (s)	Ground Truth Data		Real-Time Data	
		STFT	DMD	STFT	DMD
24,000	0.54	1.461	40.343	3.649	67.523
12,000	0.27	1.964	15.478	3.926	21.592
6000	0.13	1.478	8.950	3.794	7.474
3000	0.06	1.247	3.154	3.735	2.376
1000	0.02	1.195	1.526	3.138	1.143

**Table 6:** Specification of sensors.

Sensor Type	Specifications
Data Acquisition system	Head Lab-1 (HEAD acoustics GmbH, Herzogenrath, Germany)
Microphone	G.R.A.S 40PH Free field microphone (GRAS Sound & Vibration, Holte, Denmark)
RPM sensor	Keyence Laser sensor LV-N series (KEYENCE INDIA PVT. LTD., Chennai, India)
Accelerometer	Dytran 7583A6 Tri-axial accelerometer (Hottinger Brüel & Kjaer GmbH, Darmstadt, Germany)
In-cylinder pressure sensor	Make: Kistler, Model 6054c (Kistler Instrumente AG, Winterthur, Switzerland)

**Table 7:** Variation in signal amplitude and signal-to-noise ratio.

Distance From Source in cms	Amplitude in dB	SNR
5	76.47	31.15
10	76.27	30.77
20	76.11	29.53
30	75.82	28.30
40	74.93	27.01
55	72.65	22.97
60	70.59	20.45
70	70.36	18.97
80	69.13	17.9
90	68.94	17.8

**Table 8:** Amplitude, frequency, and decay factor of sound signals collected in the second experiment.

	Amplitude in dB	Frequency in Hz	Decay Factor	Type of Mode
Microphone 1	67.76	500	0.75	Decaying
Microphone 2	68.32	499.91	1.0008	Neutral
Microphone 3	61.17	500	1.4	Growing

**Table 9:** Ranking of pure-tone sources of a multi-sound source system.

Source	Amplitude	Frequency	Decay Factor	Type of Mode
1	72.99	499.91	0.96	Decaying
2	55.39	99.95	0.85	Decaying

**Table 10:** Relationship between various frequencies of an engine.

Parameters	Related Frequencies
Engine speed	Cam shaft frequency (0.5 × engine speed) Oil pump frequency (1 × engine speed) Coolant pump frequency (2 × engine speed)
Fundamental engine firing frequency	Piston slap frequency (third order of fundamental firing frequency)

**Table 11:** Parameters estimated from the empirical equations, calculated from the engine speed, and dominant frequencies predicted by dynamic mode decomposition (DMD) of the sound signal at 10 cm and 90 cm from the engine running at 2000RPM.

Parameters/Values in Hz	Calculated Fundamental Frequencies	Frequencies of Sound Signal at 10 cm	Frequencies of Sound Signal at 90 cm
Fundamental engine firing frequency.	50	50.1187	49.938
Piston slap frequency (third order of fundamental firing frequency)	150	149.9365	Not available
Oil pump frequency (1 × engine speed)	33.33	33.389	35.942
Coolant pump frequency (2 × engine speed)	66.66	66.79	64.5911

**Table 12:** Parameters estimated from the empirical equations, calculated from the engine speed, and dominant frequencies predicted by dynamic mode decomposition (DMD) of the sound signal at 10 cm and 90 cm from the engine running at 4250RPM.

Parameters/Values in Hz	Calculated Fundamental Frequencies	Frequencies of Sound Signal at 10 cm	Frequencies of Sound Signal at 90 cm
Fundamental engine frequency	106.25	105.97	105.85
Piston slap frequency (third order of fundamental frequency)	318.75	317.98	318.13
Oil pump frequency (1 × engine speed)	70.83	70.55	70.54
Coolant pump frequency (2 × engine speed)	141.66	141.03	Not available

**Table 13:** Sound source parameters estimated at positions 1 to 5 at the engine speed of 2000 rpm.

P_1_			P_2_			P_3_			P_4_			P_5_		
Amp.	Freq.	Decay Factor	Amp.	Freq.	Decay Factor	Amp.	Freq.	Decay Factor	Amp.	Freq.	Decay Factor	Amp.	Freq.	Decay Factor
96.45	50.04	Decaying	98.10	50.18	Decaying	95.48	50.28	Growing	94.09	49.92	Growing	93.73	49.87	Growing
87.46	33.39	Decaying	87.70	100.39	Decaying	82.49	251.20	Decaying	79.99	266.69	Decaying	80.83	3.63	Growing
84.89	100.04	Decaying	85.55	150.57	Decaying	82.00	267.65	Decaying	79.20	382.88	Decaying	79.61	266.23	Decaying
82.61	601.13	Decaying	84.89	251.03	Decaying	80.23	150.64	Decaying	77.75	865.21	Growing	78.00	398.85	Growing
77.64	589.69	Decaying	84.07	669.28	Decaying	78.15	1741.26	Neutral	77.22	284.06	Decaying	77.82	149.76	Decaying
76.82	200.26	Growing	82.72	836.68	Decaying	77.83	1157.35	Growing	77.13	635.96	Growing	77.44	233.86	Growing
76.73	549.57	Decaying	82.01	384.51	Decaying	77.58	351.50	Decaying	77.07	918.90	Neutral	77.36	698.50	Decaying
76.56	500.88	Decaying	81.00	768.29	Growing	76.78	468.95	Decaying	76.70	366.96	Growing	76.98	1177.74	Growing
76.40	686.66	Growing	80.51	501.08	Decaying	76.66	385.21	Decaying	76.58	99.92	Decaying	76.58	282.91	Growing
75.66	454.61	Decaying	80.27	685.48	Neutral	76.03	194.95	Decaying	76.56	316.67	Growing	76.30	99.60	Decaying
75.65	249.87	Decaying	80.02	66.77	Decaying	75.79	83.26	Decaying	75.65	66.77	Decaying	75.94	384.00	Growing
74.94	300.30	Growing	79.99	217.33	Decaying	75.69	318.11	Decaying	75.59	273.40	Growing	75.92	366.35	Decaying
74.66	266.94	Growing	79.91	267.36	Decaying	75.56	284.41	Decaying	75.45	880.50	Growing	75.80	1192.32	Growing
74.23	149.94	Decaying	79.42	1923.59	Neutral	75.40	1718.43	Growing	75.16	605.20	Neutral	75.28	1171.84	Decaying
74.22	216.90	Growing	78.50	602.23	Growing	75.06	842.83	Growing	75.05	1271.38	Decaying	74.95	500.83	Decaying
74.04	350.35	Decaying	78.16	592.78	Growing	74.41	857.59	Growing	75.03	926.88	Growing	74.89	249.40	Decaying
73.56	283.44	Neutral	78.02	737.02	Decaying	74.03	1695.34	Growing	75.03	647.94	Growing	74.62	977.12	Decaying
73.48	833.06	Growing	77.96	830.16	Growing	73.97	1262.20	Growing	74.89	150.08	Decaying	74.43	664.60	Growing
73.43	576.62	Neutral	77.77	1270.55	Growing	73.92	217.02	Decaying	74.81	623.04	Growing	74.42	201.56	Decaying
72.68	400.20	Decaying	76.71	885.88	Decaying	73.74	32.97	Decaying	74.49	956.63	Decaying	74.29	1379.91	Decaying
72.66	333.41	Decaying	76.64	33.76	Decaying	73.42	1251.40	Growing	74.44	599.30	Growing	74.26	616.20	Growing
72.55	509.87	Growing	76.55	1129.06	Decaying	73.22	1841.87	Growing	74.21	660.23	Growing	73.68	588.65	Neutral
72.37	66.79	Decaying	76.49	200.70	Growing	72.99	702.89	Decaying	73.98	249.53	Growing	73.64	66.58	Growing
72.10	317.07	Growing	76.02	701.07	Decaying	72.91	1201.80	Growing	73.51	698.01	Decaying	73.49	549.07	Growing
71.97	733.22	Decaying	75.89	1404.12	Growing	72.64	1292.47	Growing	73.46	300.51	Decaying	73.26	1325.50	Growing
71.74	849.57	Decaying	75.31	84.03	Growing	72.47	234.74	Decaying	73.24	1478.70	Decaying	73.13	1277.15	Neutral
71.65	632.60	Growing	75.21	1188.09	Decaying	72.34	958.13	Growing	73.09	540.09	Growing	72.89	627.09	Decaying
71.52	1433.29	Decaying	75.12	2.84	Growing	71.72	581.62	Decaying	72.56	235.04	Decaying	72.72	381.35	Growing
71.36	703.54	Decaying	75.09	307.84	Decaying	71.54	592.51	Decaying	72.53	711.94	Growing	72.25	316.02	Decaying
71.32	233.40	Growing	74.96	658.16	Growing	71.44	1676.65	Growing	72.28	1063.62	Decaying	72.19	1082.80	Growing
71.20	528.26	Decaying	73.39	753.11	Growing	71.43	1747.92	Growing	71.89	680.27	Growing	72.12	1291.08	Decaying
NA	NA	NA	73.23	167.52	Decaying	71.21	1098.62	Growing	71.44	1086.89	Decaying	72.01	658.48	Growing
NA	NA	NA	73.18	401.31	Decaying	71.18	818.66	Decaying	71.43	764.72	Growing	71.79	1613.69	Decaying
NA	NA	NA	71.00	1049.39	Growing	71.13	480.89	Decaying	71.13	588.16	Decaying	71.67	1511.71	Growing

**Table 14:** Sound source parameters estimated from the sound signal.

Mode No.	Frequency in Hz	Engine Parameter	Exists at Position
1	33.33	Oil pump frequency	1, 2, 3
1	50	Firing frequency	1, 2
1	66.66	Coolant pump frequency	1, 2, 4
2	150	Piston slap frequency	1, 2, 3, 4, 5

## Data Availability

Due to a non-disclosure agreement only ground truth data can be shared on request.

## Abbreviations

The following abbreviations are used in this manuscript:

DMD	Dynamic Mode Decomposition
FFT	Fast Fourier Transform
SNR	Signal-to-Noise Ratio
VNF	Very-Near Field
SPL	Sound Pressure Level
NAH	Near-field Acoustic Holography
STSF	Spatial Transformation of Sound Field
IBEM	Inverse Boundary Element Methods
SPC	Source Path Contribution
PNCA	Panel Noise Contribution Analysis
OTPA	Operational Transfer Path Analysis
SVD	Singular Value Decomposition
MV	Minimum Variance
MUSIC	Multiple Signal Classification
CC	Cross-Correlation
CSP	Cross-power Spectrum Phase analysis
DR	Decay Factor
STFT	Short-time Fourier Transform
MB	Mega-Byte
DAQ	Data Acquisition System
IC	Internal Combustion
RPM	Revolutions Per Minutes
